# Supplementary material for: Optimization and Characterization of a Novel Exopolysaccharide from Bacillus haynesii CamB6 for Food Applications
Source: Biomolecules. 2022 Jun 15;12(6):834. doi: 10.3390/biom12060834 (PMC9221024; doi:10.3390/biom12060834)
Supplement: Supplementary file 1 [file biomolecules-12-00834-s001.zip › biomolecules-1691308-supplementary.pdf]

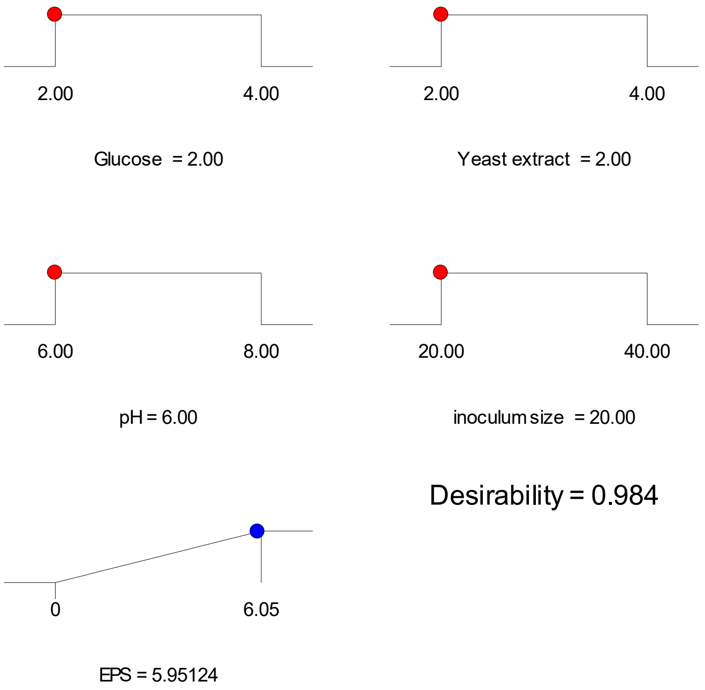

Supplementary Figure S1A. Ramp chart for EPS production

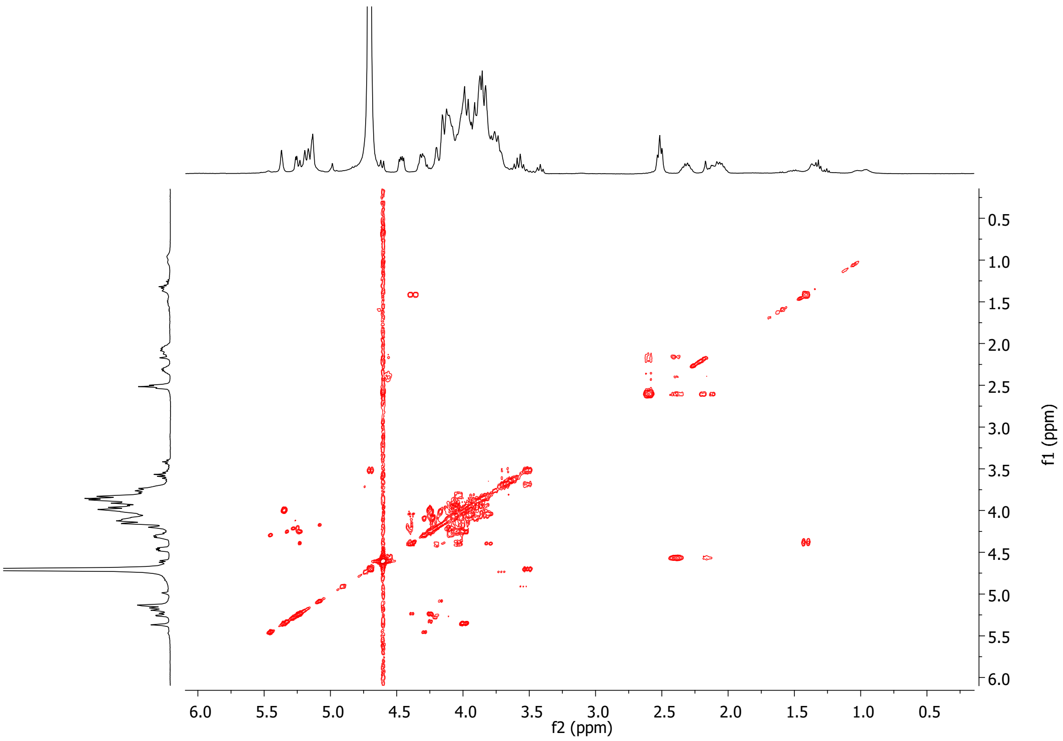

Supplementary Figure S1B. COSY spectrum of EPS produced by *B. haynesii* EPS

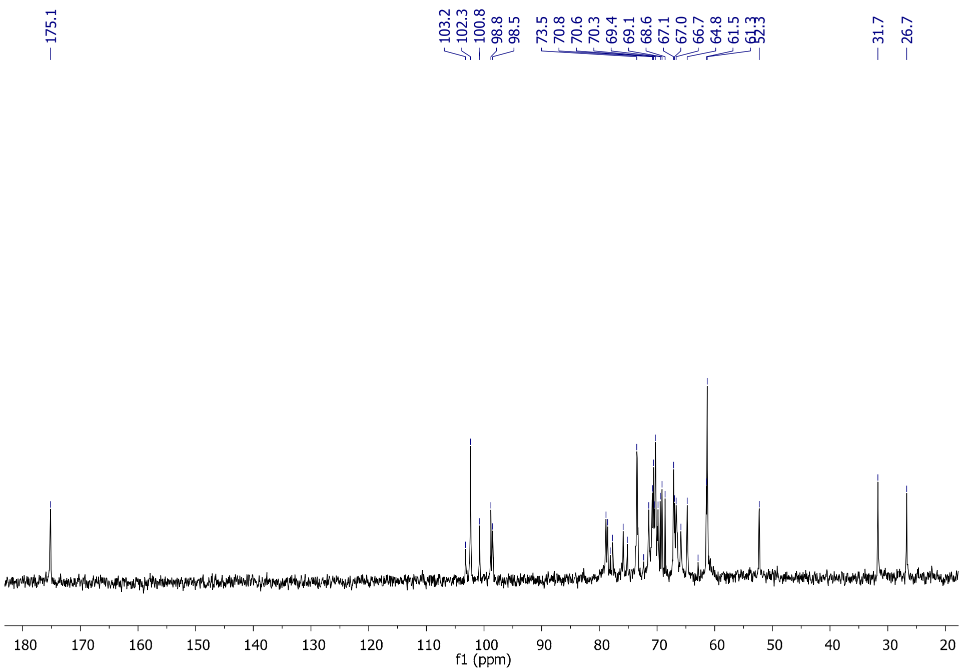

Supplementary Figure S1C. ^{13}C NMR spectrum of EPS obtained at 60°C in $^{2}H_2O$

Supplementary Table S1A: Concentration of different metals (Al, As, Cd, Cu, Cr, Fe, Mg, Mn, Hg, Ni, Pb, and Zn) (mg L^{-1} ± S.D.) (n=2) in the sample water collected from Campanario hot spring of Maule region, Chile

Al	As	Cd	Cu	Cr	Fe	Mg	Mn	Hg	Ni	Pb	Zn
0.48 ± 0.07	0.02 ± 0.00	< 0,0028	0,12 ± 0.10	0.01 ± 0.00	3.00 ± 0.43	57.70 ± 0.40	0.47 ± 0.10	< 0,00006	0.17 ± 0.02	0.24 ± 0.04	0.14 ±0.03

S.D: Standard deviation

Supplementary Table S1B: The physico-chemical parameters (n=2) of the water sample collected from Campanario hot spring of Maule region, Chile

pH	Conductivity	Alkalinity	TDS	DO	BOD	Chlorides	Sulfates	Turbidity	Color	Nitrates	TDS
	mS/cm	mg L^{-1} $CaCO_3$	mg L^{-1}	mg L^{-1}	mg L^{-1}	mg L^{-1}	mg L^{-1}	NTU	Pt/Co	mg L^{-1}	mg L^{-1}
5.83 ± 0.03	0.02 ±0.00	413.3 ±0.80	23.87 ± 0.19	9.50 ±0.30	0.65 ± 0.05	12.04 ±0.30	207.50 ± 8.70	25.70 ± 1.70	5.00 ± 0.00	4.51 ± 0.27	23.87± 0.19

S.D: Standard deviation
